# Testis-specific glyceraldehyde-3-phosphate dehydrogenase: origin and evolution

**DOI:** 10.1186/1471-2148-11-160

**Published:** 2011-06-10

**Authors:** Mikhail L Kuravsky, Vladimir V Aleshin, Dmitrij Frishman, Vladimir I Muronetz

**Affiliations:** 1 Faculty of Bioengineering and Bioinformatics, M.V. Lomonosov Moscow State University, Leninskiye Gory 1-73, Moscow 119991, Russian Federation; 2 A.N. Belozersky Institute for Physical and Chemical Biology, M.V. Lomonosov Moscow State University, Leninskiye Gory 1-40, Moscow 119991, Russian Federation; 3 Kharkevich Institute for Information Transmission Problems of the Russian Academy of Sciences, Moscow, 127994, Russian Federation; 4 Department of Genome Oriented Bioinformatics, Technische Universität München, Wissenschaftszentrum Weihenstephan, Freising 85354, Germany

## Abstract

**Background:**

Glyceraldehyde-3-phosphate dehydrogenase (GAPD) catalyses one of the glycolytic reactions and is also involved in a number of non-glycolytic processes, such as endocytosis, DNA excision repair, and induction of apoptosis. Mammals are known to possess two homologous GAPD isoenzymes: GAPD-1, a well-studied protein found in all somatic cells, and GAPD-2, which is expressed solely in testis. GAPD-2 supplies energy required for the movement of spermatozoa and is tightly bound to the sperm tail cytoskeleton by the additional N-terminal proline-rich domain absent in GAPD-1. In this study we investigate the evolutionary history of GAPD and gain some insights into specialization of GAPD-2 as a testis-specific protein.

**Results:**

A dataset of GAPD sequences was assembled from public databases and used for phylogeny reconstruction by means of the Bayesian method. Since resolution in some clades of the obtained tree was too low, syntenic analysis was carried out to define the evolutionary history of GAPD more precisely. The performed selection tests showed that selective pressure varies across lineages and isoenzymes, as well as across different regions of the same sequences.

**Conclusions:**

The obtained results suggest that GAPD-1 and GAPD-2 emerged after duplication during the early evolution of chordates. GAPD-2 was subsequently lost by most lineages except lizards, mammals, as well as cartilaginous and bony fishes. In reptilians and mammals, GAPD-2 specialized to a testis-specific protein and acquired the novel N-terminal proline-rich domain anchoring the protein in the sperm tail cytoskeleton. This domain is likely to have originated by exonization of a microsatellite genomic region. Recognition of the proline-rich domain by cytoskeletal proteins seems to be unspecific. Besides testis, GAPD-2 of lizards was also found in some regenerating tissues, but it lacks the proline-rich domain due to tissue-specific alternative splicing.

## Background

Glyceraldehyde-3-phosphate dehydrogenase (GAPD, EC 1.2.1.12) is a homotetrameric glycolytic enzyme providing phosphorylation of 3-phosphoglyceraldehyde to 1,3-diphosphoglycerate coupled with reduction of NAD^+ ^to NADH. Mammals are known to possess two tissue-specific GAPD isoenzymes: somatic (GAPD-1) and testis-specific (GAPD-2, GAPDS). For *Homo sapiens*, their protein sequences are 68% identical. Besides the two isoenzymes, a vast amount of GAPD pseudogenes was found in the genomes of primates and rodents [1,2].

Mammalian GAPD-1 is a well-studied protein, a high concentration of which in cells (5-15% of all cytoplasmic proteins) confirms its functional significance. Recent studies established that GAPD-1 is not simply a classical metabolic protein involved in glycolytic energy production, but rather a multifunctional protein with specific functions in numerous processes [3,4]. GAPD-1 was shown to display both cytosolic and nuclear localization participating in endocytosis [5-7], plasma membrane fusion [8], microtubule assembly [9,10], secretory vesicular transport [11,12], protein phosphotransferase/kinase reactions [13,14], translational and transcriptional controls of gene expression [15-17], regulation of telomere structure [18,19], nuclear membrane fusion [20], nuclear RNA transport [21], DNA excision-repair [22,23] and induction of apoptosis in case of oxidative stress [24-27]. Furthermore, GAPD-1 was implicated in Alzheimer's [28-30] and Huntington's [30-32] neurodegenerative diseases.

As opposed to soluble GAPD-1, mammalian GAPD-2 is tightly attached to the cytoskeleton, namely to the principal piece of the spermatic filament fibrous sheath [33-35]. The attachment is mediated by an additional N-terminal proline-rich domain of 74 amino acids [35,36]. GAPD-2 supplies the dynein ATPases of filament with energy, therefore playing a crucial role in the maintaining of sperm motility. Disruption of its expression generally leads to infertility [37]. Due to its strong association with cytoskeleton GAPD-2 remains within the insoluble fraction after cell breaking, significantly complicating its experimental investigation. As a result, there is only little data on GAPD-2 properties. It was recently discovered to display enhanced stability towards denaturation that may be an adaptation to the absence of protein expression in spermatozoa. Enzyme kinetics exhibited by GAPD-2 was found to differ from the one exhibited by GAPD-1 too [38]. Based on the study of short functional motives of both mammalian isoenzymes, GAPD-2 was proposed to evade involvement in most non-glycolytic processes characteristic for GAPD-1 [39].

GAPD-1 and GAPD-2 are also possessed by some other vertebrates besides Mammalia [40-42], but their expression is apparently not always tissue-specific. In the bony fish *Oplegnathus fasciatus *both GAPD mRNAs were detected ubiquitously in all of tissues examined [40], and therefore the functional specificities of the isoenzymes seem to differ from the mammalian ones. Based on the phylogenetic trees, it was hypothesized that GAPD could diverge to the isoenzymes around the origin of Bilateria, but as only vertebrates have retained GAPD-2, this scenario seems unlikely. However, some vertebrates (e.g. *Xenopus laevis*) were discovered to lack GAPD-2 [42].

Single copy genes are thought to evolve conservatively because of strong negative selective pressure. Gene duplications produce a redundant gene copy and thus release one or both copies from negative selective pressure. Thus, duplications should be an important precursor of functional divergence. The increased availability of sequences in the public databases allows the investigation of the molecular evolution of the GAPD gene family and the evaluation of selection following duplication events. In the present study we focus on the evolution of the poorly uninvestigated GAPD-2 isoenzyme. Previously GAPD-2 was discovered to be specific for vertebrates [42]. Therefore we will focus on this taxon as well as on the other groups of deuterostomes not considered in [42]. Specifically, we (1) examine the evolutionary history of GAPD-2 and other GAPD isoenzymes of deuterostomes, (2) evaluate lineage-specific changes in selective pressure affecting GAPD isoenzymes, and (3) look into the metamorphosis of GAPD-2 to a testis-specific protein.

## Results

### Sequences of GAPD family members

**Figure 1:**
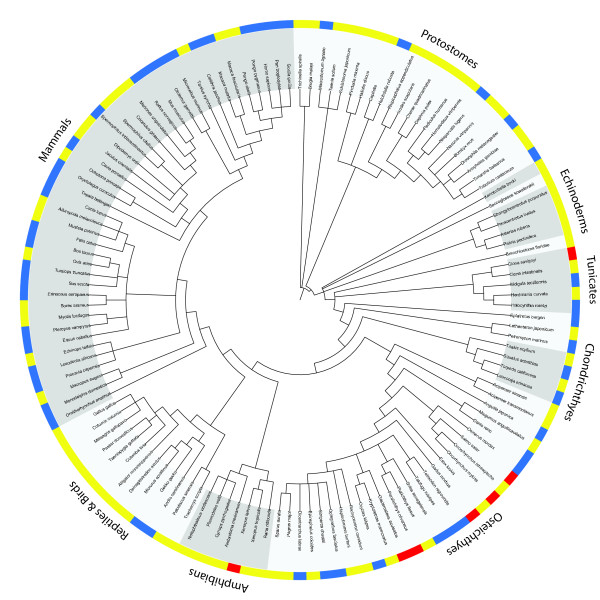
**Numbers of predicted GAPD genes for the examined species**. Yellow color corresponds to species with a single predicted gene, blue color - with two genes and red - with three genes. Taxonomy was obtained from NCBI Taxonomy database [119]; the figure was prepared with iTOL [120].

The numbers of discovered GAPD family members for all examined species are represented at Figure 1. Mammalian GAPD sequences were extracted from the Ensembl database. For most species (19 of the 25 examined) two different sequences were obtained. One of these sequences always contained an additional proline-rich domain at the N-terminus, as observed in the human GAPD-2. A single GAPD sequence was obtained for each of the 6 remaining mammalian species, either with or without the proline-rich domain. The lack of the second sequence seems to be due to incompleteness of genomes.

GAPD sequences of teleosts were obtained using both Ensembl (5 species present in this database) and BLAST searches against RefSeq transcripts and the EST division of GenBank (species not covered by Ensembl). Three different sequences were discovered for 4 species, two sequences - for 6 species and a single sequence - for 3 species. The differences between the numbers of obtained GAPD sequences are not necessarily a result of data incompleteness and may be biologically relevant. For example, only two sequences were identified within a complete genome of zebrafish.

Identification of GAPD sequences of all other species was performed by conducting BLAST searches against RefSeq transcripts and the EST division of GenBank. Two different GAPD sequences were discovered for lizards, some cartilaginous fishes, some jawless vertebrates, some tunicates and a few non-deuterostomes (3 of 10 insects, a leech and a flatworm). Single GAPD sequences were discovered for all examined birds, reptiles except lizards, amphibians, lancelet, echinoderms, acorn worm, *Xenoturbella bocki*, as well as for the remaining cartilaginous fishes, jawless vertebrates, tunicates and most examined non-deuterostomes. Two species (*Xenopus laevis *and *Ciona savignyi*) were revealed to possess even three, but slightly different GAPD family members.

### Tissue-specific translation of the proline-rich domain in lizard GAPD

**Figure 2:**
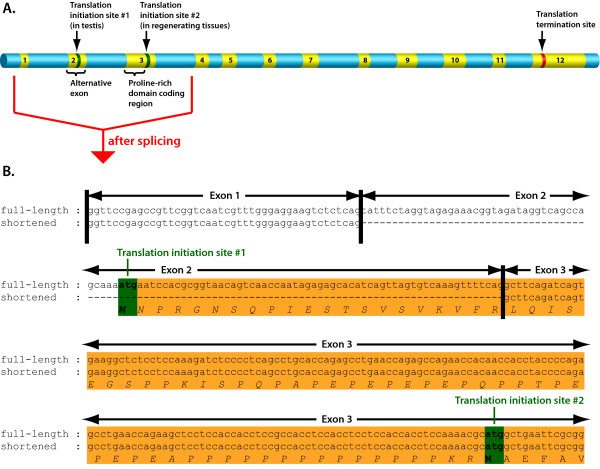
**Alternative splicing in GAPD-2 of *Anolis carolinensis***. Alternative splicing seems to govern the proline-rich domain presence in GAPD-2 of a lizard *Anolis carolinensis*. If the second exon is spliced, the protein product will lack the proline-rich domain, otherwise it will possess this domain. A) Map of GAPD-2 gene constructed based on both Ensembl and EST data. Exons are in yellow, introns and not-transcribed regions are in blue. The positions of the two possible translation initiation sites are marked, as well the position of the translation termination site. B) Alignment of the 5'-termini of full-length and shortened (lacking the second exon) mRNAs. The sequences of possible protein products are also represented.

Besides mammalian GAPD-2, proline-rich domains were detected only in one of the GAPD isoenzymes of lizard species: *Anolis carolinensis *and *Gekko gecko*. ESTs of *A. carolinensis *encoding this isoenzyme originated from testis [GenBank:FG786985, GenBank:FG793471, GenBank:FG801901, GenBank:FG802958], regenerating tail [GenBank:FG771974, GenBank:FG779496] and the whole embryo [GenBank:FG720854]. It is remarkable that ESTs from regenerating tail and embryo lack a fragment of 103 nucleotides (shortened variant), which in present in ESTs from testis (full-length variant; see Figure 2 and additional file 1: Alignment of the two forms of Anolis carolinensis GAPD-2 mRNA). This fragment is situated near the 5'-terminus and encodes the beginning of the proline-rich domain including an ATG start codon. The next possible start codon, which is present in both EST variants, is located right after the proline-rich domain. So the protein with the proline-rich domain should be translated only from the full-length EST variant. Translation of the shortened EST variant should begin from the second start codon such that the product will not possess the proline-rich domain.

Availability of the two EST variants must be a result of tissue-specific alternative splicing: the exon of 103 nucleotides was either preserved, as in gonads, or spliced out, as in embryo and regenerating tail. Thus, the presence of the proline-rich domain has a tissue-specific character.

A few ESTs of *G. gecko *were extracted from samples of injured brain and spinal cord [GenBank:EB170778, GenBank:CV053413] and had incomplete 5'-termini: only a part of the sequence encoding the proline-rich domain was present. Therefore it is impossible to ascertain whether the translation of the proline-rich domain in *G. gecko *is governed by alternative splicing like in *A. carolinensis*.

### Phylogeny and syntenic analyses

**Figure 3:**
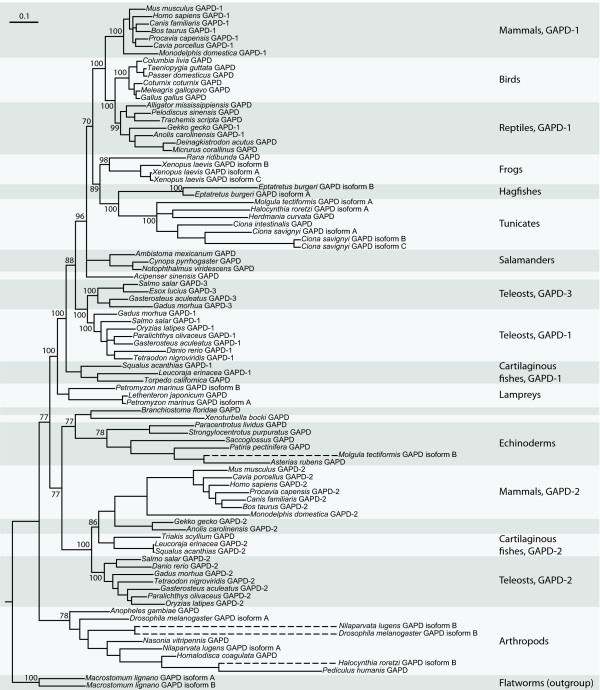
**Phylogenetic tree of 92 GAPD isoenzymes**. Phylogenetic tree constructed on nucleotide sequences using the Bayesian algorithm. Numbers at nodes are the obtained posterior probabilities. Discontinuous lines mark the branches of enormous high length (more than 0.75), which can correspond to pseudogenes or contaminated samples. The tree does not fit the common knowledge about the evolution in details; nevertheless it provides some useful information. For more accurate definition of GAPD evolution, syntenic analysis was also used.

Analysis of the orthologous and paralogous relationships of GAPD isoenzymes among different species was carried out by combining the phylogeny reconstruction of the GAPD gene family with syntenic comparison. The phylogenetic tree constructed from amino-acid sequences demonstrated poor correspondence to the common knowledge about the evolution of deuterostomes, probably due to high sequence conservation (only 48 of 335 residues are different between GAPD-1 of human and its ortholog in zebrafish). Therefore we decided to switch to nucleic sequences which are less conserved. Indeed, the obtained phylogenetic tree (Figure 3) showed better correspondence to the common evolutionary knowledge, but still was far from perfect. For example, tunicates were closer to mammals than fishes.

All GAPD isoenzymes of vertebrates can be subdivided into two groups based on the clades of phylogenetic tree: the group including mammalian GAPD-1 and the group including mammalian GAPD-2. GAPD of insects separates before these two groups diverge, which means that the duplication into GAPD-1 and GAPD-2 took place after the divergence of protostomes and deuterostomes. The orthologs of mammalian GAPD-1 and GAPD-2 are further referred to as GAPD-1 and GAPD-2, correspondingly.

The clade including mammalian GAPD-1 is supported by a high posterior probability (100%). Inside this clade a number of additional duplications were detected. One of them apparently happened near the origin of teleosts and produced a third GAPD isoenzyme hereinafter referred to as GAPD-3. Other independent duplications produced additional GAPD isoenzymes in lamprey, hagfish, sea squirt and *Xenopus laevis*.

The clade including mammalian GAPD-2 is based on a less robust branch with a posterior probability of 77%. It splits into a clade of vertebrates (100% posterior probability) and a clade including the only GAPD isoenzymes of echinoderms, lancelet, hemichordates and *Xenoturbella bocki*, as well as the second GAPD isoenzyme of some tunicates (77% posterior probability). On account of lower support value, merging of these two clades into one is questionable and needs confirmation.

**Figure 4:**
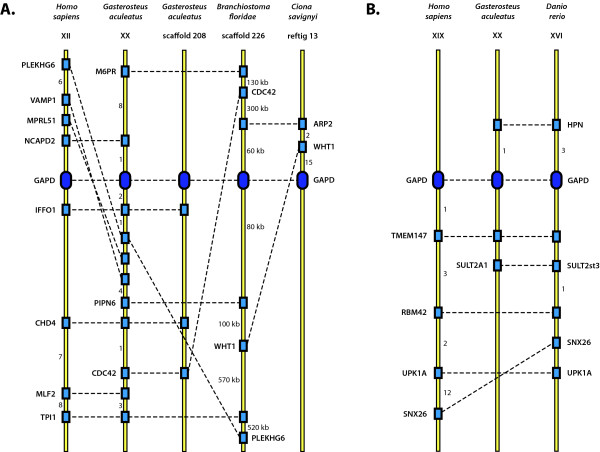
**Synteny maps**. Syntenic comparison of GAPD genes among human (*Homo sapiens*), stickleback (*Gasterosteus aculeatus*), zebrafish (*Danio rerio*), lancelet (*Branchiostoma floridae*) and sea squirt (*Ciona savignyi*). A) The locus containing GAPD-1 of human, GAPD-1 of stickleback, GAPD-3 of stickleback, the only GAPD isoenzyme of lancelet and one of GAPD isoenzymes of sea squirt. B) The locus containing GAPD-2 of human, GAPD-2 of stickleback and GAPD-2 of zebrafish. GAPD genes are shown by ovals, other genes - by rectangles. Homologues are indicated by discontinuous lines. The numbers near the yellow axes mean either the quantities of genes which are not shown or the distances in kilobases.

The syntenic analysis showed that GAPD family members of the examined species can be linked to either of two loci: the locus syntenic to human GAPD-1 contains GAPD-1 of zebrafish, GAPD-1 and GAPD-3 of stickleback, the only GAPD of lancelet and the only GAPD of sea squirt; the locus syntenic to human GAPD-2 contains GAPD-2 of both zebrafish and stickleback (Figure 4). The similarity between gene layouts within both loci is rather low, multiple genome micro-rearrangement events such as deletions and inversions were detected. The surroundings of GAPD genes in sea urchin and acorn worm genomes do not contain any common genes with both each other and the revealed two syntenic loci. The genes in these surroundings do not form any clusters in the genomes of other examined species as well. This can be accounted for distant relationships between the species.

BLAST searches of genes which are syntenic to human and fish GAPD-2 were carried out in the genomes of lancelet, sea squirt, sea urchin and acorn worm. They showed that these genes are dispersed in the genomes rather than combined together in a single locus.

The constructed synteny maps provide support for orthology between GAPD-1 of human, either GAPD-1 or GAPD-3 of stickleback, GAPD of lancelet and sea squirt, as well as between GAPD-2 of human and both fishes. These results generally agree with the phylogenetic trees, indicating orthology between appropriate isoenzymes of human and fishes. Syntenic analysis helped to identify the origin of lancelet and sea squirt GAPDs, which was not determined with confidence by phylogenetic trees construction because of low branch support values. The evidence is also given for the origination of GAPD-1 and GAPD-3 of stickleback and probably some other bony fishes as a result of teleost-specific whole genome duplication.

### Selective pressure estimation

K_a_/K_s _profiles were compared in four clades: mammalian GAPD-1 and GAPD-2, teleost GAPD-1 and GAPD-2, while GAPD of insects was used as an outgroup. To avoid saturation in synonymous substitutions which can significantly affect the results, pairs of closely related sequences were considered (Table 1).

**Table 1:** Pairs of sequences used for K_a_/K_s _calculation

Taxon	Species	Isoenzyme	Sequence identity, %
Mammals	*Homo sapiens - Microcebus murinus*	GAPD-1	91

	*Homo sapiens - Callitrix jacchus*	GAPD-2	94

Teleosts	*Tetraodon nigroviridis - Takifugu rubripes*	GAPD-1	94

	*Tetraodon nigroviridis - Takifugu rubripes*	GAPD-2	92

Insects	*Drosophila ananassae - Drosophila virilis*	GAPD	87

**Figure 5:**
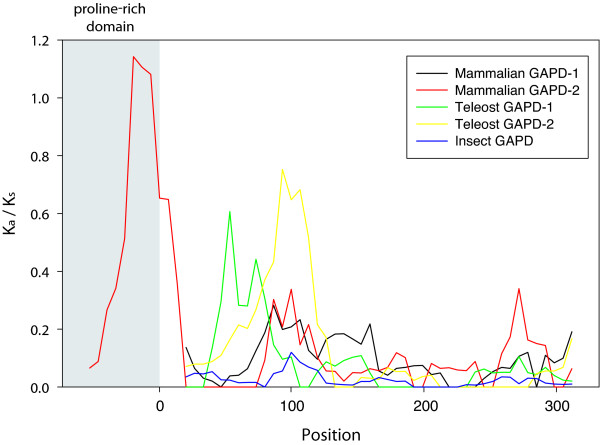
**K_a_/K_s _profiles**. Five GAPD isoenzymes are compared: GAPD-1 and GAPD-2 of mammals, GAPD-1 and GAPD-2 of teleosts and GAPD of insects. Numbering of the X axis starts immediately after the proline-rich domain of mammalian GAPD-2 and corresponds to the positions in protein sequences.

Results of K_a_/K_s _profile calculation show that selective pressure varies for different regions of GAPD sequences (Figure 5). Most regions of all examined sequences are suggested to be under strong purifying selection (K_a_/K_s _< 0.1). However, a part of the proline-rich domain of mammalian GAPD-2 is not restrained by purifying selection with K_a_/K_s _up to 1.1.

In mammalian GAPD-1 and GAPD-2, teleost GAPD-2 and insect GAPD, the purifying selection is impaired approximately between the 85th and 105th positions of protein sequences (Section 1; from here on the numbering of amino acid positions as in mammalian GAPD-1). In mammalian GAPD-2 purifying selection is also weakened between the 265th and 285th positions (Section 2). In teleost GAPD-1 purifying selection is weakened between the 55th and 75th positions (Section 3).

The regions under impaired purifying selection were mapped on the 3D-structure of human GAPD-1 (PDB ID 1u8f). Section 1 corresponds to a buried β-strand and adjacent loops near the NAD-binding site. Sections 2 and 3 are solvent-exposed regions of the polypeptide chain also composed of both β-strands and loops.

Selective pressure affecting GAPD family members was also investigated by means of branch-specific models as implemented in PAML. Six datasets were examined: mammalian GAPD-1 (17 sequences) and GAPD-2 (12 sequences), teleost GAPD-1 (10 sequences), GAPD-2 (7 sequences) and GAPD-3 (4 sequences) as well as insect GAPD (8 sequences). To determine whether the selective constrains vary for different isoenzymes and lineages, two models were compared: one-ratio (R1) and six-ratios (R6). R1 assumed constant K_a_/K_s _ratio for all examined GAPD datasets, whereas R6 assumed different ratios for each dataset. The obtained K_a_/K_s _ratios and the likelihoods of the models are represented in Table 2. The likelihood ratio test (LRT) indicated a significant difference between the likelihoods of R1 and R6 (2d = 148.57, df = 5, p-value = 0.00), implying variation of selective constrains at least for some datasets.

**Table 2:** The K_a_/K_s _ratio estimates for GAPD isoenzymes under various branch-specific models

Model	K_a_/K_s _ratio	Log-likelihood
R1	0.06369	-22221.41

R2m	0.05363 (all except mammalian GAPD-2) 0.12179 (mammalian GAPD-2)	-22179.54

R2i	0.07405 (all except insect GAPD) 0.02642 (insect GAPD)	-22177.37

R3	0.06263 (all except listed below) 0.12186 (mammalian GAPD-2) 0.02567 (insect GAPD)	-22150.49

R6	0.06672 (mammalian GAPD-1) 0.05218 (fish GAPD-1) 0.07492 (fish GAPD-3) 0.12187 (mammalian GAPD-2) 0.06218 (fish GAPD-2) 0.02593 (insect GAPD)	-22147.12

**Figure 6:**
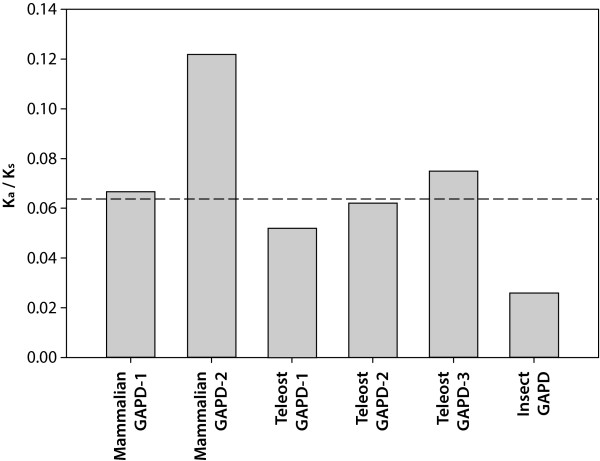
**K_a_/K_s _values obtained with the aid of branch-specific models**. The shown K_a_/K_s _values were calculated using the six-ratio (R6) model, which implies different selection constrains for GAPD-1 and GAPD-2 of mammals, GAPD-1, GAPD-2 and GAPD-3 of teleosts, as well as GAPD of insects. The values for all isoenzymes except mammalian GAPD-2 and insect GAPD were found not to differ significantly. Discontinuous horizontal line is the K_a_/K_s _value obtained using the one-ratio (R1) model, which implies the same selection constrains for all isoenzymes.

Following the results obtained for R6 model, the K_a_/K_s _ratios of mammalian GAPD-2 and insect GAPD differ from the mean value above all (Figure 6). Therefore the hypotheses stating that the selective constrains differ between these two and the other datasets were tested. Three models were compared with R6: R2m model assuming constant K_a_/K_s _ratio for all datasets except mammalian GAPD-2, R2i model assuming constant K_a_/K_s _ratio for all datasets except insect GAPD and R3 model assuming constant K_a_/K_s _ratio for all datasets except both mammalian GAPD-2 and insect GAPD (see Table 2 for the obtained ω-values and likelihoods). LRT revealed that the likelihoods of R3 and R6 are not significantly different (2d = 6.73, df = 3, p-value = 0.08), while the likelihoods of both R2m and R2i are significantly lower (2d = 64.84, df = 4, p-value = 0 and 2d = 60.5, df = 4, p-value = 0, respectively). It means that the selective constrains are more or less similar for all three teleost GAPD isoenzymes and mammalian GAPD-1, greater for insect GAPD and weaker for mammalian GAPD-2.

## Discussion

### Evolutionary relationships between GAPD isoenzymes

In this study we sought to expand the previous phylogenetic investigations of GAPD [42-50] by concentrating on deuterostomes. As compared to the study in reference [42], which is also focused on deuterostomes, we introduced a number of new sequences especially from non-mammalian and non-teleost species and carried out the syntenic analysis. This allowed more accurate determination of phylogeny, as well as the identification of some novel GAPD isoenzymes, for example the third isoenzyme of teleosts.

**Figure 7:**
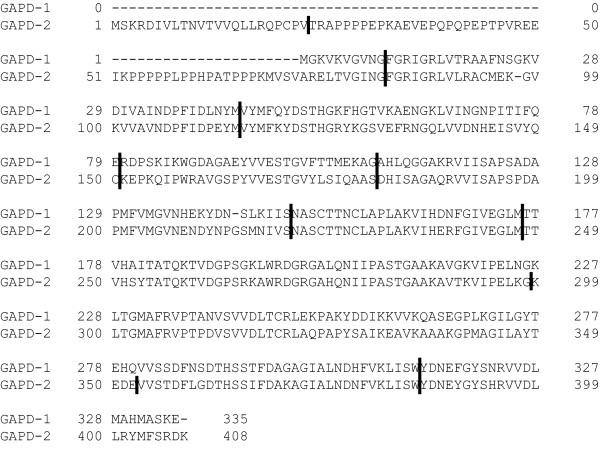
**Exon structures of human GAPD-1 and GAPD-2**. The boundaries of exons are shown by vertical lines. The figure is based on Ensembl data [96].

The constructed phylogenetic trees provide evidence for duplication in the early evolution of chordates which gave rise to GAPD-1 and GAPD-2 isoenzymes. It presumably took place even before the first whole-genome duplication of vertebrates [51-53]. The loci of GAPD-1 and GAPD-2 were found not to be syntenic to each other. It can be explained either by a single-gene duplication, which produced a copy of the ancestral GAPD gene, or by loss of synteny after a duplication of longer genome segment. However, the emergence of GAPD-1 and GAPD-2 is surely not a result of a retroposition, as it was concluded in early studies [54,55], documented by similar exon structures of the isoenzymes (Figure 7). It should be noted that GAPD is one of the few glycolytic enzymes that did not acquire any additional isoenzymes during the vertebrate-specific whole-genome duplication events; neither did phosphoglucose isomerase, triosephosphate isomerase and phosphoglycerate kinase. The other glycolytic enzymes gained from one to three extra copies that evolved to the tissue-specific proteins [42,56-61].

GAPD-2 was lost in most lineages and retained only by mammals, lizards, teleosts and cartilaginous fishes. The presence of both isoenzymes in these organisms raises the question of a functional difference between them. It is assumed that if two isoenzymes perform the same function in the same set of tissues, one of them is free from functional constraints and its gene will eventually turn into a non-functional pseudogene or will be deleted [62-64]. In mammals and lizards GAPD-1 and GAPD-2 specialized to tissue-specific proteins and this is probably the reason why one of them avoided the lost. Generally, specialization towards tissue-specificity is a trend among glycolytic enzymes that have acquired additional copies. In vertebrates, they usually have distinctive isoenzymes in liver, muscle and brain, sometimes in erythrocytes and other tissues [42,60]. The situation with GAPD of teleosts and cartilaginous fishes is more complex. According to EST data, GAPD-1 and GAPD-2 of fishes are expressed in the same tissues. The results of branch-specific tests indicate that the evolutionary rates of both isoenzymes are accelerated as compared to the ancestral GAPD (GAPD of insects, which separated before the emergence of GAPD-1 and GAPD-2, was considered to evolve with the similar rate as the ancestral protein). This is in line with the model of gene duplications proposed by Hughes [65,66]. It suggests that the original gene was performing two or more functions. After duplication each copy specialized on performing a part of them. GAPD is known to be a multifunctional protein participating in many processes beyond glycolysis. As the catalytic center is conserved in both isoenzymes, GAPD-1 and GAPD-2 of teleosts and cartilaginous fishes may specialize on performing different non-glycolytic functions, as also evidenced by K_a_/K_s _profiling. Different regions of teleost GAPD-1 and GAPD-2 are under impaired purifying selection. These regions can correspond to the parts of proteins which are responsible for performing isoenzyme-specific non-glycolytic functions.

A number of additional duplications of GAPD genes occurred independently in certain lineages. For example, some teleosts possess the third GAPD isoenzyme (GAPD-3) in addition to GAPD-1 and GAPD-2. Taking into account both the constructed phylogenetic trees and the obtained data on syntenies, it can be concluded that GAPD-3 originated from GAPD-1 during the teleost-specific whole-genome duplication [67]. However, GAPD-3 was not found in complete genomes of zebrafish, tetraodon and fugu, which means that it was lost.

The retention of GAPD-3 by certain species of teleosts agrees with the model of dosage balance proposed by Papp with colleagues [65,68]. It states that genes having optimal dosages that are dependent on each other may be lost only synchronously after whole-genome duplications. Therefore they are preferentially kept. In the study by [42] most of the other glycolytic enzymes were shown to have extra copies in teleosts, which also originated during the whole-genome duplication. Therefore, GAPD-3 as well as the other additional glycolytic isoenzymes in teleosts may be retained to prevent dosage imbalance leading to glycolysis malfunction.

The model of dosage balance also provides an explanation for Xenopus laevis possessing three slightly different GAPD isoenzymes [Swiss-Prot:P51469, GenBank:BC043972, GenBank:BC048770]. Following the results of phylogenetic analysis (Figure 3), the duplications of GAPD-1 gene giving rise to these isoenzymes seem to have taken place after the divergence of Xenopus and Rana genera of frogs. X. laevis is known to have undergone a whole-genome duplication event about 40 million years ago [69,70] and most of its genes have two copies [71]. Furthermore, the GAPD genes in reference [GenBank:BC043972, GenBank:BC048770] might be the allelic variants of a single gene since they are 99% identical and their evidence is only at transcript level. If so, X. laevis would have only two GAPD genes, in line with the dosage balance model.

Sea squirt Ciona savignyi was discovered to possess three different GAPD isoenzymes as well. All of them seem to have originated from GAPD-1 after the emergence of tunicates (Figure 3). To check whether these isoenzymes are encoded by distinct genes or allelic variants of a single gene, we turned to the C. savignyi genome assembly version 2.0 (Broad Institute) with removed redundant alleles [72] available via Ensembl. There were only two GAPD genes [Ensembl:ENSCSAVG00000004357, Ensembl:ENSCSAVG00000007442] corresponding to the two isoenzymes. The remaining isoenzyme was 97% identical to one of the others. Perhaps, it is nothing but an allelic variant since C. savignyi displays extremely high allelic polymorphism [73].

It looks like the duplication giving rise to the GAPD-1 copies in C. savignyi is advantageous itself by increasing GAPD dosage. Otherwise fixation of even two consecutive duplications seems to be unlikely. The model of gene duplications assuming beneficial increase in gene dosage has been extensively studied and shown to be applicable in a number of cases [74-76]. The duplications of GAPD in the considered species may be explained by the emerged necessity of enhancing of some non-glycolytic functions of GAPD, as it is hard to imagine that such a conserved process as glycolysis needs an increase of a dose of one of its enzymes.

### GAPD-2 specialization to a testis-specific protein

Mammalian GAPD-2 is known to be a highly specialized isoenzyme, which is present solely in testis (microarray data are available in the ArrayExpress database at http://www.ebi.ac.uk/arrayexpress under accession numbers E-GEOD-7307, E-GEOD-3526, E-TABM-969 and E-GEOD-2361) [77-79]. We have found that GAPD-2 is expressed in a testis-specific way also by two lizard species. Lizards are also the only lineage besides mammals in which GAPD-2 possesses the proline-rich domain. Taking into account that this domain serves as an anchor to spermatic filament cytoskeleton, correlation between its presence and testis-specific expression seems to be evident.

As GAPD-2 is a testis-specific protein only in mammals and lizards, it is likely to have specialized in this way during the early evolution of amniots. However, birds have completely lost GAPD-2. We could not detect it in any of the examined bird species including *Gallus gallus *and *Taeniopygia guttata *with complete genomes. So, the same GAPD isoenzyme should act in both somatic tissues and testis. It remains unclear what changes in bird spermatozoa rendered testis-specific GAPD-2 unnecessary.

GAPD-2 is not the only testis-specific glycolytic isoenzyme. There are also testis-specific isoenzymes of phosphoglycerate kinase (PGK-2) [80,81] and lactate dehydrogenase (LDHC) [82-84]. It is remarkable that both are possessed only by mammals, thus resembling GAPD-2. PGK-2 originated from PGK-1 isoenzyme by retrotransposition [85,86], while LDHC stems from the LDHA isoenzyme [60]. These events are supposed to have taken take place during the early evolution of mammals. Perhaps, the gain of three testis-specific glycolytic isoenzymes is a consequence of an alteration of spermatozoa structure. Mammalian spermatozoa are known to have a relatively long and thin tail, complicating ATP diffusion from mitochondria along it [87]. Therefore, energy is generated mostly by glycolytic enzymes located in the tail cytoplasm [34,88]. Such reorganization of metabolism may require special isoenzymes with distinctive catalytic properties.

As mentioned before, a unique feature of testis-specific GAPD-2 is the additional N-terminal proline-rich domain, which is absent in all other GAPD isoenzymes. Moreover, there are no additional fragments in PGK-2 and LDHC. The spatial structure of the proline-rich domain is still unsolved. We have found that for the majority of mammals it is encoded by two exons. The first exon encodes a conservative segment of 22 amino acids. The second exon encodes a segment with a high content of proline residues, highly variable in both length (58-97 amino acids) and composition (see additional file 2: The proline-rich N-terminal domains of mammalian GAPD-2). The layout of proline residues has a strikingly repetitive character. They form P_n _and (XP)_n _motifs, where X is any amino acid (often cysteine, glutamic acid or glutamine). Generally, polyproline repetitive motifs are known to participate in strong but unspecific protein-protein interactions [89]. Apparently they play the same role in the proline-rich domain of GAPD-2 mediating the binding to spermatic filament cytoskeleton. The presence of two different kinds of polyproline motifs suggests GAPD-2 being bound to more than one protein of cytoskeleton.

An evidence for unspecific proline-rich domain recognition by cytoskeletal proteins is also furnished by the results of K_a_/K_s _calculation. K_a_/K_s _value estimated for the variable segment of the proline-rich domain of mammalian GAPD-2 was close to unity, which means that this domain is subjected to neither purifying nor positive selection and therefore its specific sequence is not important for functioning.

The proline-rich domain is likely to be relatively young since it is absent in all other GAPD isoenzymes and no similar sequences have been revealed in other proteins by means of BLAST searches. So-called exonization of non-coding sequences is now assumed to be the source of new protein domains [90-93]. The repetitive character of the proline-rich domain sequence implies that it could have emerged from a microsatellite region. This way of new domain origination was proposed to be a general mechanism for the repetitive protein sequences [90,94,95].

Tissue-specific alternative splicing was discovered to govern the presence of proline-rich domain in GAPD-2 of a lizard *Anolis carolinensis*: it depends on a cassette exon being either spliced or retained. Unfortunately, no conclusion can be made as to whether this mechanism preceded GAPD-2 specialization to a testis-specific protein or appeared after it. It may be that GAPD-2 first incorporated the proline-rich domain as a rare optional splice variant in some tissues and only then specialized towards testis-specificity.

## Conclusions

The results of our study substantially expand the current knowledge on evolution of GAPD family members. We show that GAPD-1 and GAPD-2 isoenzymes of mammals are also present in other lineages. We speculate that they emerged after duplication of the ancestral GAPD gene during the early evolution of chordates. GAPD-1 then underwent a number of additional independent duplications in different species, while GAPD-2 was lost in most lineages and is now found only in mammals and lizards, as well as cartilaginous and bony fishes.

We have demonstrated that GAPD-2 of mammals and lizards is specialized to a testis-specific protein. Accordingly, in these lineages GAPD-2 has acquired the novel N-terminal proline-rich domain anchoring the protein to the sperm tail cytoskeleton. This domain is likely to have originated by exonization of a microsatellite genomic region in a common ancestor of amniots. Estimates of selective pressure suggest unspecific recognition of the proline-rich domain by cytoskeletal proteins. Besides testis, GAPD-2 of lizards was also found in some regenerating tissues, but lacking the proline-rich domain due to tissue-specific alternative splicing.

## Methods

### Sequence data

In the previous study [42], GAPD-2 was shown to be specific for vertebrates. Therefore we decided to limit the consideration of GAPD isoenzymes and focused only on those belonging to vertebrates and also to the other groups of deuterostomes since they were not examined in [42]. In order to find all GAPD sequences of deuterostomes, we first turned to the Ensembl database [96]. 69 sequences of mammals and bony fishes were obtained from it as belonging to glyceraldehyde-3-phosphate dehydrogenase protein family [Ensembl:ENSFM00250000000211]. Second, a PSI-BLAST [97] search using the human GAPD-1 [SwissProt:P04406] as query (which was selected to be a typical example of GAPD) was conducted against UniProt [98]. Since GAPD is known to be a well-conserved protein, a strict e-value threshold of 10^-6 ^was chosen. The search converged in 6 steps returning 8957 hits, all of which showed more than 30% of identity to the query sequence. All in all 60 sequences of deuterostomes were picked out (excluding fragments and those previously obtained from the Ensembl database). We also selectively picked out 13 sequences of the major protostome phyla (arthropods, mollusks, annelid worms, roundworms and flatworms). Third, additional 55 sequences were obtained by employing TBLASTN algorithm with default parameters [97] to search with human GAPD-1 [SwissProt:P04406] and GAPD-2 [SwissProt:O14556] as queries in the EST division of GenBank [99]. EST hits, which usually represent fragments of complete mRNAs, were manually scanned for extensive overlapping regions and then joined into larger sequences. Further inspection revealed some cases of contamination, which were excluded from the analysis. Specifically, we identified a chicken EST [GenBank:AM067846] actually belonging to *Aspergillus flavus *and three lancelet ESTs [GenBank:FE567488, GenBank:FE567489, GenBank:BW781185] belonging to some diatoms. As a result of this three step procedure the total of 197 GAPD sequences were identified for 131 species (109 deuterostomes and 22 other animals, see additional file 3: Accession codes of GAPD sequences used in the analysis).

### Multiple alignment and phylogeny reconstruction

Since phylogenetic tree reconstruction is a computationally expensive process, only a part of the obtained sequences was subjected to the analysis. No more than 7 species from each class of deuterostomes were considered, as well as 6 species of insects as the representatives of protostomes (for more details see additional file 3: Accession codes of GAPD sequences used in the analysis). Two slightly different GAPD sequences from the flatworm *Macrostomum lignano*, both derived from several ESTs [GenBank:EG952499, GenBank:EG951174, GenBank:EG952414, GenBank:EG952720, GenBank:EG953822], were used as an outgroup. The total dataset for phylogenetic analysis comprised 92 GAPD sequences. Multiple alignment of protein sequences was performed by MUSCLE [100] and then manually edited. The alignment of nucleic sequences was constructed by means of RevTrans 1.4 Server [101] based on the protein alignment (see additional file 4: Raw alignment of GAPD nucleic sequences used in the phylogenetic analysis). Columns with gaps were eliminated before phylogenetic analysis.

The phylogenetic relationships between GAPD family members were reconstructed using both protein and nucleic sequences. The Bayesian method of tree reconstruction as implemented in MrBayes 3.1.2 [102,103] software was applied. The JTT model of amino-acid change [104], as well as the GTR model of nucleotide substitutions [105] were used. Preliminary analyses indicated that variation at the third position was saturated and confounded resolution at deep internal nodes. Therefore, trees based on nucleotide data were reconstructed in MrBayes by partitioning the data into the first, second and third codon positions, and allowing each partition to evolve at its own rate with its own shape parameter of gamma distribution.

For the Bayesian analyses, two independent runs were performed, each with four simultaneous chains that sampled every 100 generation. Trees sampled before the cold chain reached stationarity based on plots of the maximum likelihood scores were discarded. Sampling continued until convergence was achieved based on the average standard deviation of the split frequencies as given in MrBayes. Node support was accessed as Bayesian posterior probabilities.

### Syntenies

Syntenic analysis is a reliable approach for establishing orthology. It is based on the assumption that local surroundings of genes are rarely affected by genomic rearrangements. Therefore, if the two genes have homologous neighbors, they are likely to have originated by vertical descent from a single ancestor and, in other words, be orthologous.

A syntenic analysis of the relationship between GAPD family members was performed by identification of positions of up to 20 genes both upstream and downstream of GAPD genes in human (*Homo sapiens*), stickleback (*Gasterosteus aculeatus*), zebrafish (*Danio rerio*), lancelet (*Branchiostoma floridae*), sea squirt (*Ciona savignyi*), sea urchin (*Strongylocentrotus purpuratus*) and acorn worm (*Saccoglossus kowalevskii*). Syntenic maps were constructed based on the information regarding gene location either available from Ensembl (human, both fishes and sea squirt) or obtained by conducting BLASTX searches of adjacent genomic regions against non-redundant protein databases. In the latter case, the homology between genes was decided if the identities of their protein product sequences were greater than 30%. The following genomes were used: B. floridae version 2.0 (Joint Genome Institute) [51], S. purpuratus version 2.1 (Human Genome Sequencing Center) [106] and S. kowalevskii version 1.0 (Human Genome Sequencing Center). Since genomic micro-rearrangements might occur, the matches between the local surroundings of GAPD genes were not required to be co-linear for establishing orthology. Gene losses and insertions were allowed as well.

### Synonymous and non-synonymous substitution rates

To examine whether the GAPD family members are subjected to adaptive evolution, an analysis of variation under selective pressure was performed. Usually selective pressure is estimated by comparing the rates of synonymous (K_s_) and non-synonymous substitutions (K_a_) for the entire sequence. If Ka/Ks value is greater than unity, the whole sequence is supposed to be under positive selection, otherwise under purifying selection [107-109]. However, since each amino acid has a different function, the type and strength of natural selection may be different for each amino acid. To detect the variation in K_a_/K_s _values across the sequence a sliding-window approach is often used [110,111].

Alignments of nucleotide sequences were constructed by PAL2NAL [112] based on protein alignments. K_a_/K_s _profiles were generated using a window of 120 base pairs and a step of 20 base pairs. Such a wide window was used because of high conservation of the analyzed sequences. Calculations of K_a _and K_s _for each window position were carried out with the aid of DnaSP 5.10 software [113].

### Branch-specific selection tests

The differences in selective pressure between GAPD isoenzymes were also examined by means of more sophisticated branch-specific models as implemented in codeml program from PAML software [114]. Such kind of models assumes separate Ka/Ks values for different branches of the phylogenetic tree. They are often used for detecting selection changes after gene duplications, where one copy might evolve at a different rate due to acquisition of a new function or the loss of an old one [115-118].

First, GAPD sequences were divided into groups according to the results of phylogenetic analysis. Then a number of branch-specific models assuming separate K_a_/K_s _ratios for different combinations of groups were assayed. Likelihood ratio test (LRT) was used to determine whether the likelihoods of a pair of alternative branch-specific models are significantly different.

## Authors' contributions

MLK participated in collection of sequences, construction of phylogenetic trees, syntenic analyses and performing selection tests, and drafted the manuscript. VVA participated in collection of sequences, construction of phylogenetic trees, syntenic analyses, and performing selection tests. DF helped to collect sequences, participated in performing selection tests, and helped write the manuscript. All authors read and approved the final manuscript. VIM conceived the study, participated in its design and coordination, gained funding and helped to draft the manuscript.

## Supplementary Material

Additional file 1 **Alignment of the two forms of *Anolis carolinensis *GAPD-2 mRNA**. The sequence of protein product is represented; the possible start codons are in bold. Translation of the full-length mRNA seems to begin from the first start codon and the protein product will possess the proline-rich domain (shown in italic). The shortened mRNA lacks the first start codon. Therefore translation should begin from the second one resulting in a protein product without the proline-rich domain. Click here for file

Additional file 2 **The proline-rich N-terminal domains of mammalian GAPD-2**. Click here for file

Additional file 3 **Accession codes of GAPD sequences used in the analysis**. Click here for file

Additional file 4 **Raw alignment of GAPD nucleic sequences used in the phylogenetic analysis**. Click here for file
