# Supplementary material for: Testis-specific glyceraldehyde-3-phosphate dehydrogenase: origin and evolution
Source: BMC Evol Biol. 2011 Jun 10;11:160. doi: 10.1186/1471-2148-11-160 (PMC3224139; doi:10.1186/1471-2148-11-160)
Supplement: Additional file 1 — Alignment of the two forms of Anolis carolinensis GAPD-2 mRNA. The sequence of protein product is represented; the possible start codons are in bold. Translation of the full-length mRNA seems to begin from the first start codon and the protein product will possess the proline-rich domain (shown in italic). The shortened mRNA lacks the first start codon. Therefore translation should begin from the second one resulting in a protein product without the proline-rich domain. [file 1471-2148-11-160-S1.PDF]

```
full-length : ggttccgagccgttcggtcaatcgtttgggaggaagtctctcagtatttctaggtagagaaacggtagataggtcagccagcaaaatgaatccacgcggtaacagtcaaccaatagagag
shortened   : ggttccgagccgttcggtcaatcgtttgggaggaagtctctc------------------------------------------------------------------------------
                                                                                                                M  N  P  R  G  N  S  Q  P  I  E  S

full-length : cacatcagttagtgtcaaagttttcaggcttcagatcagtgaaggctctcctccaaagatctcccctcagcctgcaccagagcctgaaccagagccagaaccacaaccacctaccccaga
shortened   : -------------------------aggcttcagatcagtgaaggctctcctccaaagatctcccctcagcctgcaccagagcctgaaccagagccagaaccacaaccacctaccccaga
               T  S  V  S  V  K  V  F  R  L  Q  I  S  E  G  S  P  P  K  I  S  P  Q  P  A  P  E  P  E  P  E  P  E  P  Q  P  P  T  P  E

full-length : gcctgaaccagaagctcctccaccacctccgccacctccacctcctccaccacctccaaaacgcatggctgaattcgcggttggaatcaatggatttggacgcattggccgcttggtgct
shortened   : gcctgaaccagaagctcctccaccacctccgccacctccacctcctccaccacctccaaaacgcatggctgaattcgcggttggaatcaatggatttggacgcattggccgcttggtgct
               P  E  P  E  A  P  P  P  P  P  P  P  P  P  P  P  P  P  P  K  R  M  A  E  F  A  V  G  I  N  G  F  G  R  I  G  R  L  V  L

full-length : ccgagcctgcgtcgagaagggtatcaaagtggtagcaatcaatgacccgttcattgacctcaactatatggtctatatgttcaagtatgattccactcatggacgtttcaaaggagtggt
shortened   : ccgagcctgcgtcgagaagggtatcaaagtggtagcaatcaatgacccgttcattgacctcaactatatggtctatatgttcaagtatgattccactcatggacgtttcaaaggagtggt
               R  A  C  V  E  K  G  I  K  V  V  A  I  N  D  P  F  I  D  L  N  Y  M  V  Y  M  F  K  Y  D  S  T  H  G  R  F  K  G  V  V

full-length : gaaggccgatggtggcaaattagtagtgaatgacttaaaaatatctgtgttccagtgcatgaaaccaactgagattccttggggagatgcaggagctctgtatgtagtggagtctaccgg
shortened   : gaaggccgatggtggcaaattagtagtgaatgacttaaaaatatctgtgttccagtgcatgaaaccaactgagattccttggggagatgcaggagctctgtatgtagtggagtctaccgg
               K  A  D  G  G  K  L  V  V  N  D  L  K  I  S  V  F  Q  C  M  K  P  T  E  I  P  W  G  D  A  G  A  L  Y  V  V  E  S  T  G

full-length : agtcttcctttctgttgacaaagcttcagctcatctgtctggtggagccaggcgagtagtggtgactgcaccatctcctgatgctcctatgtttgttatgggagtcaatcatgagaagta
shortened   : agtcttcctttctgttgacaaagcttcagctcatctgtctggtggagccaggcgagtagtggtgactgcaccatctcctgatgctcctatgtttgttatgggagtcaatcatgagaagta
               V  F  L  S  D  D  K  A  S  A  H  L  S  G  G  A  R  R  V  V  V  T  A  P  S  P  D  A  P  M  F  V  M  G  V  N  H  E  K  Y

full-length : tgatccagactccatgaatgttctgagtaatgcttcctgcactactaactgcctggcacctttggccaaagtcattcacgacaactttggcattgtggagggccttatgaccactgttca
shortened   : tgatccagactccatgaatgttctgagtaatgcttcctgcactactaactgcctggcacctttggccaaagtcattcacgacaactttggcattgtggagggccttatgaccactgttca
               D  P  D  S  M  N  V  L  S  N  A  S  C  T  T  N  C  L  A  A  D  P  D  S  M  N  V  L  S  N  A  S  C  T  T  N  C  L  A  A

full-length : tgcttatactgccactcagaagactgtagatggtccctctgccaaggcctggcgagatgggagaggggcccaccagaacatcatcccagcttctactggtgctgccaaagcagtgggcaa
shortened   : tgcttatactgccactcagaagactgtagatggtccctctgccaaggcctggcgagatgggagaggggcccaccagaacatcatcccagcttctactggtgctgccaaagcagtgggcaa
               A  Y  T  A  T  Q  K  T  V  D  G  P  S  A  K  A  W  R  D  G  R  G  A  H  Q  N  I  I  P  A  S  T  G  A  A  K  A  V  G  K

full-length : ggccatccctgagcttaatgggaagctcactggcatggcattccgtgtgccagtgccagatgtctcggttgttgacctgacatgccgcctctccaggccagccacctatgctcagatcaa
shortened   : ggccatccctgagcttaatgggaagctcactggcatggcattccgtgtgccagtgccagatgtctcggttgttgacctgacatgccgcctctccaggccagccacctatgctcagatcaa
               A  I  P  E  L  N  G  K  L  T  G  M  A  F  R  V  P  V  P  D  V  S  V  V  D  L  T  C  R  L  S  R  P  A  T  Y  A  Q  I  K

full-length : agacgctatcaagaaggcacctaaggggccaatggcaggaatcctaggatatacagaagatgaggttgcttctactgatttcattggtgacaatcgttcctccatctttgatgctggagc
shortened   : agacgctatcaagaaggcacctaaggggccaatggcaggaatcctaggatatacagaagatgaggttgcttctactgatttcattggtgacaatcgttcctccatctttgatgctggagc
               D  A  I  K  K  A  P  K  G  P  M  A  G  I  L  G  Y  T  E  D  E  V  A  S  T  D  F  I  G  D  N  R  S  S  I  F  D  A  G  A

full-length : aggaatctccctgaatgataactttgtgaagctcatttcctggtatgataatgaatatggctacagctgccgtgtagcagatctcctgaagcacatgtacaccaaggagcacaactga
shortened   : aggaatctccctgaatgataactttgtgaagctcatttcctggtatgataatgaatatggctacagctgccgtgtagcagatctcctgaagcacatgtacaccaaggagcacaactga
               G  I  S  L  N  D  N  F  V  K  L  I  S  W  Y  D  N  E  Y  G  Y  S  C  R  V  A  D  L  L  K  H  M  Y  T  K  E  H  N  *
```
